# Possible transmission of human immunodeficiency virus-1 infection from an elite controller to a patient who progressed to acquired immunodeficiency syndrome: a case report

**DOI:** 10.1186/1752-1947-6-291

**Published:** 2012-09-11

**Authors:** Michael Scott Killian, Girish N Vyas, Rochak Mehta, Karen Young, Osman Ebrahim

**Affiliations:** 1 Department of Medicine, University of California San Francisco, 513 Parnassus Avenue, Box 1270, San Francisco, CA, 94143, USA; 2 Department of Laboratory Medicine, University of California San Francisco, 513 Parnassus Avenue, Box 0134, San Francisco, CA, 94143, USA; 3 Roche Molecular Diagnostics, 4300 Hacienda Drive, Pleasanton, CA, 94588, USA; 4 Department of Medical Microbiology, Faculty of Health Sciences, University of Pretoria, Pretoria, South Africa

## Abstract

**Introduction:**

Most individuals infected with human immunodeficiency virus-1, in the absence of antiretroviral therapy, exhibit persistent virus replication and declining CD4+ cell numbers, and progress to acquired immunodeficiency syndrome within 10 years of infection. Elite controllers are rare individuals with human immunodeficiency virus-1 infection who can maintain undetectable plasma virus levels and remain asymptomatic without antiretroviral therapy. It has been proposed that elite controllers benefit from being infected with attenuated human immunodeficiency virus-1 variants.

**Case presentation:**

A 31-year-old African woman presented with human immunodeficiency virus-1 infection during pregnancy and was diagnosed with acquired immunodeficiency syndrome. Subsequently, her husband, a 31-year-old African man, was tested and found to be seropositive for human immunodeficiency virus-1. His plasma human immunodeficiency virus-1 ribonucleic acid level was found to be below the limit of detection of the clinical assay.

**Conclusion:**

This report provides evidence for the first described case of human immunodeficiency virus-1 infection possibly transmitted from an elite controller to a patient who progressed to acquired immunodeficiency syndrome. This observation strengthens the case against avirulence as a mechanism that protects elite controllers.

## Introduction

Most individuals with human immunodeficiency virus-1 (HIV-1) infection who are untreated have plasma viral loads that exceed 1000 HIV-1 ribonucleic acid (RNA) copies/mL and they progress to acquired immunodeficiency syndrome (AIDS) within 10 years of infection [1]. Some people infected with HIV-1 maintain undetectable viral loads for years, and can remain asymptomatic for indefinite periods of time (>20 years) [2]; these individuals are known as elite controllers (ECs). The biologic mechanism(s) for their remarkable control of HIV-1 replication has not been determined [3]. The transmission of HIV-1 infection from a patient with AIDS to an EC has been documented [4]. This finding was not unexpected as HIV-1 infection is most likely to be transmitted by individuals having high viral loads, such as those with untreated AIDS [5-7]. Here, we provide evidence for a possible reciprocal case of HIV-1 transmitted from an EC to a patient who then progressed to AIDS.

## Case presentation

Patient FF presented as a 31-year-old pregnant woman from Johannesburg, who was tested for HIV and found to be HIV-1 seropositive 24 hours before delivery by Caesarean section in 2001. At that time, she was promptly given a dose of nevirapine to prevent perinatal transmission. Further clinical and laboratory testing revealed that FF had AIDS. Her peripheral blood cluster of differentiation CD4+ cell count was 61cells/μL (7%) and her plasma viral load was 51,700 HIV-1 RNA copies/mL. She had florid oral candidiasis and was emaciated. Since initiating combination antiretroviral therapy consisting of zidovudine, lamivudine and efavirenz, her viral load has reached an undetectable level (<20 HIV-1 RNA copies/mL) and her CD4+ cell count has rebounded to 435 cells/μL. FF confirmed that, prior to her marriage to her husband (MM) in 2000, she had tested negative for HIV-1 during a previous pregnancy and that child, from a previous partner, was also negative for the virus. That child was breastfed by FF for at least 12 months and remained uninfected. Our patient reported that since 2000, her husband is the only partner with whom she has had sexual contact.

Patient MM, the husband of FF, presented as a 31-year-old man for HIV-1 testing soon after his wife was found to be HIV-1 seropositive. In an interview, MM stated that he had been monogamous in his marital relationship with FF and that he had no prior sexual partners. MM gave no history of any infectious diseases but had undergone surgery three years previously for treatment of an abdominal gunshot wound. He reported having received a blood transfusion in association with that surgery. At that time in South Africa, the screening for HIV-1 contamination of blood for transfusion was not as thorough as present standards (polymerase chain reaction (PCR)-based screening of blood for HIV-1 contamination was being newly implemented in South Africa in 2000; GNV, independent observation). On examination, MM was found to be generally healthy, with vital signs and examination of all systems being normal. MM was found to be HIV-1 seropositive by Western blot. However, he had an undetectable plasma viral load (<50 HIV-1 RNA copies/mL), his CD4+ T cell count of 1123/μL (32%) was well within the normal range, and his CD4+ T cell to CD8+ T cell ratio was not inverted. In accordance with standard clinical guidelines at that time, no antiretroviral treatment was initiated.

**Figure 1:**
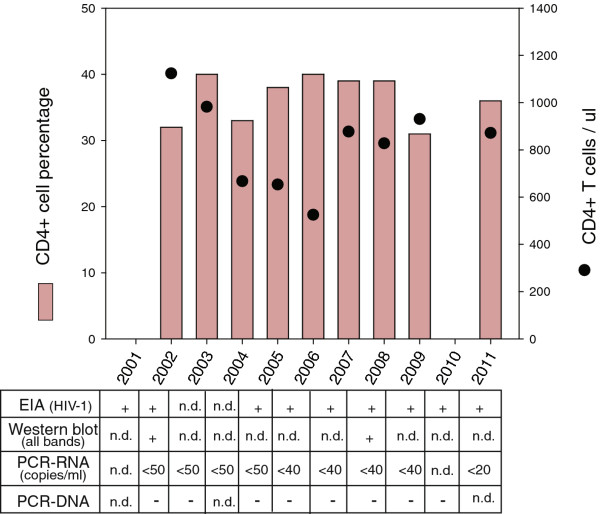
**Longitudinal measurements of CD4+ cells and human immunodeficiency virus-1 levels. **During a period of 10 years, CD4+ cell percentages and absolute counts were monitored in blood from patient MM. Also performed were measurements of human immunodeficiency virus-1 ribonucleic acid, deoxyribonucleic acid, and p24 levels and anti-human immunodeficiency virus-1 antibodies. EIA, enzymatic immunoassay; (+), positive test result; (-), negative test result; n.d., test not performed; HIV-1, human immunodeficiency virus-1; PCR-RNA, polymerase chain reaction-ribonucleic acid; PCR-DNA, polymerase chain reaction-deoxyribonucleic acid.

During a follow-up period of 10 years (Figure 1), MM consistently maintained an undetectable (<50 HIV-1 RNA copies/mL) plasma viral load. Over this 10 year period, MM tested negative for HIV-1 deoxyribonucleic acid (DNA) by PCR on at least eight separate occasions. His CD4+ cell counts (525 to 1123 cells/μL) and percentages (31% to 40%) stayed in the normal range. He remained asymptomatic, free of the opportunistic infections and cancers that characterize AIDS.

To compare their viruses, peripheral blood mononuclear cells (PBMC) were isolated upon Ficoll gradient separation of whole blood specimens that were collected from MM and FF in 2008. Total nucleic acids (both DNA and RNA) were extracted from the PBMC, and HIV-1 sequences were reverse transcribed, amplified by nested-PCR and then sequenced at Roche Molecular Diagnostics (Pleasanton, CA, USA). Noteworthy is that this successful effort to obtain HIV-1 DNA from MM involved more intensive efforts (for example, isolation of PBMC, extraction of nucleic acids from increased numbers of CD4+ T cells, and nested-PCR) than the standard procedures used for the qualitative HIV-1 DNA measurements shown in Figure 1.

**Figure 2:**
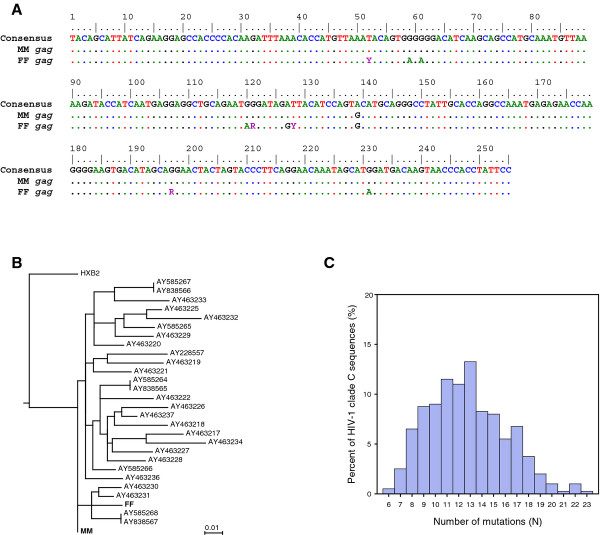
**Analysis of the patients’ human immunodeficiency virus-1 sequences*. ***A highly-conserved 255 base pair region of the human immunodeficiency virus-1 *gag *gene was sequenced. (**A**) Shown are the nucleotide sequences obtained from FF and MM, as compared with the consensus human immunodeficiency virus-1 clade C sequence. Standard ambiguity codes are used (R = G or A; Y = T or C). (**B**) The human immunodeficiency virus-1 *gag* sequences from MM and FF, along with 27 human immunodeficiency virus-1 clade C sequences from South Africa, were aligned and then analyzed with the PhyML tool [8]. Shown is a phylogenetic tree made with the maximum likelihood method. The human immunodeficiency virus-1 clade B HXB2 *gag* sequence is included as an outgroup. GenBank accession numbers are given for the comparison sequences. (**C**) The human immunodeficiency virus-1 *gag* sequence from MM was compared with other human immunodeficiency virus-1 clade C viruses published in the Los Alamos human immunodeficiency virus-1 database using the QuickAlign tool [8]. Shown is a frequency histogram of the published clade C sequences based on the number of nucleotides that differ from the MM human immunodeficiency virus-1 *gag* sequence.

Analyses were performed on a highly-conserved 255 base pair region of the HIV-1 *gag* (p24) gene (bases 1305 to 1559 in HXB2) (Figure 2). The *gag* sequences from MM and FF (Figure 2A) were compared with other HIV-1 sequences in the Los Alamos HIV database [8]. Phylogenetic analysis revealed that both sequences clustered with HIV-1 clade C viruses (data not shown), a subtype that accounts for roughly half of the HIV infections worldwide [9]. Using the maximum likelihood procedure [10], the *gag* sequences of MM and FF were found to cluster together in comparison with 27 other HIV-1 clade C *gag* sequences from South Africa (Figure 2B). In comparison with the consensus HIV-1 clade C sequence, MM differed at one of the 255 bases, while FF differed at six of the 255 bases. Importantly, the one difference in MM’s sequence was present in FF’s sequence. Thus, the *gag* sequences from MM and FF differed at only five nucleotide positions. Further analyses, performed using the QuickAlign tool in the HIV database, revealed that the majority of published clade C sequences differ from MM’s sequence in this region at greater than 10 of 255 nucleotides (Figure 2C). Moreover, less than 1% of the 400 published clade C viruses differed at six or fewer bases in this region. Even fewer would be expected to share only the discrepant nucleotide. These data demonstrate that the HIV-1 sequences from MM and FF are uniquely similar and provide evidence for the possibility that HIV-1 was transmitted between MM and FF.

## Discussion

It has not been previously appreciated that ECs can transmit HIV-1 infection. In this report, we present evidence for the possible transmission of HIV-1 from an EC to another individual. The primary evidence for this event is the medical history of MM involving reasonable risk for HIV-1 infection, the clinical history of FF, the self-reported sexual histories of MM and FF, and comparisons of the HIV-1 sequences found in MM and FF.

A limitation of this case report is its reliance on self-reported histories for risk of HIV-1 infection; we trust that MM and FF have provided truthful HIV-risk histories. Thus, it remains possible that we are incorrect in our assessment of the transmission history. Other scenarios include the transmission of HIV-1 from FF to MM. This sequence of events would discount the presumption that MM was infected with HIV-1 upon blood transfusion, but would allow for FF having a more typical AIDS-free survival time [11]. Even so, phylogenic analyses cannot prove that the transmission of HIV-1 occurred directly between two individuals [12-14]. In this regard, prospective studies of HIV-1 transmission among sero-discordant couples, with the partner infected with HIV-1 being an EC, are needed.

We were not able to determine the clinical stage when MM possibly transmitted HIV-1 to FF. Most cases of HIV-1 infection are thought to be transmitted during acute infection, a period of three to six weeks following initial infection that features the highest viral loads of any stage of HIV-1 infection [15]. Little is known about acute HIV-1 infection in ECs and whether or not ECs exhibit high viral loads at this stage remains to be determined. Nonetheless, the case we present raises the concern that HIV-1 can possibly be transmitted in the absence of detectable plasma virus levels.

## Conclusions

This case study details evidence for the possible transmission of HIV-1 infection from an EC to his wife. Emphasized is that apathogenic HIV-1 in one individual can cause typical disease in another. Efforts to control the spread of HIV-1 infection should take into consideration the potential roles of aviremic individuals in transmitting the virus.

## Consent

Written informed consent was obtained from the patients for publication of this case report and accompanying images. A copy of the written consent is available for review by the Editor-in-Chief of this journal.

## Abbreviations

AIDS: acquired immune deficiency syndrome; DNA: deoxyribonucleic acid; EC: elite controllers; HIV-1: human immunodeficiency virus 1; PBMC: peripheral blood mononuclear cells; PCR: polymerase chain reaction; RNA: ribonucleic acid.

## Competing interests

The authors declare that they have no competing interests.

## Authors’ contributions

SK participated in the virologic studies and sequence analyses, drafted the manuscript, and prepared the figures. GV participated in the virologic studies, coordinated the study, and helped to draft the manuscript. RM performed the virus sequencing assays. KY participated in the virus sequencing assays and helped to draft the manuscript. OE was the lead clinician, interviewed the patients, obtained signed informed consent forms, coordinated the clinical assays, and helped draft the manuscript. All authors read and approved the final manuscript.
